# Cytotoxic Nitrogenous Terpenoids from Two South China Sea Nudibranchs *Phyllidiella pustulosa*, *Phyllidia coelestis*, and Their Sponge-Prey *Acanthella cavernosa*

**DOI:** 10.3390/md17010056

**Published:** 2019-01-16

**Authors:** Qihao Wu, Wen-Ting Chen, Song-Wei Li, Jian-Yu Ye, Xia-Juan Huan, Margherita Gavagnin, Li-Gong Yao, Hong Wang, Ze-Hong Miao, Xu-Wen Li, Yue-Wei Guo

**Affiliations:** 1 State Key Laboratory of Drug Research, Shanghai Institute of Materia Medica, Chinese Academy of Sciences, Zuchongzhi Road 555 Zhangjiang Hi-Tech Park, Shanghai 201203, China; qihaowu@zjut.edu.cn (Q.W.); windychen_simm@163.com (W.-T.C.); simmswli@163.com (S.-W.L.); 3150103114@zju.edu.cn (J.-Y.Y.); huanxj@simm.ac.cn (X.-J.H.); yaoligong@simm.ac.cn (L.-G.Y.); zhmiao@simm.ac.cn (Z.-H.M.); 2 College of Pharmaceutical Sciences, Zhejiang University of Technology, Hangzhou 310014, China; hongw@zjut.edu.cn; 3 Consiglio Nazionale delle Ricerche (CNR), Istituto di Chimica Biomolecolare (ICB), Via Campi Flegrei, 34, 80078 Pozzuoli (Na), Italy; mgavagnin@icb.cnr.it

**Keywords:** nitrogenous terpenoids, South China Sea, sponge, nudibranch, cytotoxicity

## Abstract

A detailed chemical investigation of two South China Sea nudibranchs *Phyllidiella pustulosa* and *Phyllidia coelestis*, as well as their possible sponge-prey *Acanthella cavernosa*, led to the isolation of one new nitrogenous cadinane-type sesquiterpenoid xidaoisocyanate A (**1**), one new naturally occurring nitrogen-containing kalihinane-type diterpenoid bisformamidokalihinol A (**16**), along with 17 known nitrogenous terpenoids (**2**–**15**, **17**–**19**). The structures of all the isolates were elucidated by detailed spectroscopic analysis and by the comparison of their spectroscopic data with those reported in the literature. In addition, the absolute stereochemistry of the previously reported axiriabiline A (**5**) was determined by X-ray diffraction (XRD) analysis. In a bioassay, the bisabolane-type sesquiterpenoids **8**, **10**, and **11** exhibited cytotoxicity against several human cancer cell lines.

## 1. Introduction

Sea slugs of the genus *Phyllidiella* and *Phyllidia* are prolific in the South China Sea. They are well known for their ability to ingest toxic nitrogenous sesquiterpenoids from their diets, and use either these metabolites themselves or their biosynthetically transformed derivatives as a weapon for chemical defense [1,2,3,4,5,6,7]. An intriguing ecological study showed that when sea slugs are under attack, they release a lot of mucus containing these nitrogenous metabolites to poison their enemies [8]. The dietary origin of nitrogenous sesquiterpenoids has been supported by chemical investigations involving the isolation of such metabolites from both nudibranchs and their sponge-preys [9,10,11,12,13].

Marine sponges of the genus *Acanthella* are well known as a rich source of diverse diterpenoids and sesquiterpenoids containing nitrogenous functional groups, including cyano, isocyano, isothiocyano, and formamido functionalities [14,15,16,17,18]. Many of these secondary metabolites merit further investigation due to their various biological activities ranging from cytotoxic [15], antimalarial [19,20], and antimicrobial [21,22] to antifouling properties [14,23,24,25,26,27]. Some of them, with novel structures and promising biological activities, have attracted much attention from chemists seeking to perform their total synthesis in parallel with intensive biological studies towards new drug leads [28,29,30,31].

In our previous chemical investigation on South China Sea (Hainan) nudibranchs and sponges, nitrogenous terpenoids were isolated and structurally characterized [1,17,18,32,33,34]. In the course of our continuing project on searching for chemically fascinating and biologically active secondary metabolites from Hainan marine molluscs, as well as the chemical ecology study between nudibranchs and their sponge-preys, we made different collections of two nudibranchs, *Phyllidiella pustulosa* and *Phyllidia coelestis*, as well as their sponge-prey *Acanthella cavernosa*, from the same location (Xidao Island, Hainan Province, China), with the aim of accumulating their nitrogenous metabolites for further study of their bioactivities, as well as studying the dietary relationship between *P. pustulos*, *P. coelestis*, and their sponge-prey *A. cavernosa*.

## 2. Results

**Figure 1:**
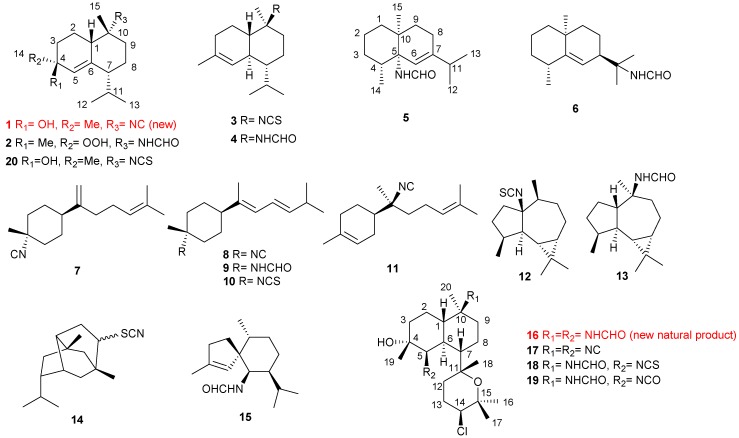
Structures of compounds **1**–**20**.

Chemical investigation of the collected two nudibranchs, *P. pustulosa* and *P. coelestis*, as well as one sponge, *A. cavernosa*, led to the isolation of one new cadinane-type sesquiterpenoid (**1**), one new naturally occurring kalihinane-type diterpenoid (**16**), along with 14 known sesquiterpenoids (**2**–**15**) and three known diterpenoids (**17**–**19**) (Figure 1). All the compounds contain nitrogen atoms in different functional groups, such as isocyanate, isothiocyanate, and formamide. Herein, we describe the isolation, structure elucidation, and cytotoxic activity of these compounds, as well as their possible biosynthetic origin influenced by the prey-predator relationship.

### 2.1. Phyllidiella pustulosa

The Et_2_O soluble portion of the acetone extract of the mollusc *P. pustulosa* was subjected to silica gel chromatography (petroleum ether/ether gradient). Guided by NMR analysis, the selected terpene-containing fractions were subsequently purified on repeated column chromatography (silica gel, Sephadex LH-20, reversed phase-C18 and RP-HPLC) to afford one new cadinane-type sesquiterpenoid (**1**), along with nine known metabolites (**2**, **3**, **6**–**8**, **12**–**14**, **17**) (Figure 1). The known compounds were identified as two cadinane-type sesquiterpenoids: halichon G (**2**) [35] and 10-isothiocyanato-4-cadinene (**3**) [13,28,36,37,38], one eudesmane-type sesquiterpenoid: 11-formamido-7*β*H-eudesm-5-ene (**6**) [39,40], two bisabolane-type sesquiterpenoids: Δ^7,14^-3-isocyanotheonellin (**7**) [1,41] and 3-isocyanotheonellin (**8**) [1], two aromadendrane-type sesquiterpenoids: 1-isothiocyanatoaromadendrane (**12**) [42] and axamide-2 (**13**) [43,44], one mixture of pupukeanane-type sesquiterpenoids: 9-thiocyanatopupukeanane isomers (**14**) [6], and one kalihinane-type diterpenoid: kalihinol A (**17**) [45].

Compound **1**, namely xidaoisocyanate A, was obtained as a colorless oil, ${\left[\alpha \right]}_{D}^{20}$ −3.6 (*c* 0.1, MeOH). Its molecular formula, C_16_H_25_NO, was established by HREIMS (*m*/*z* 247.1927, [M]^+^, calcd. 247.1936), indicating five degrees of unsaturation (Figures S1 and S2). The diagnostic ^1^H and ^13^C NMR resonances, as well as coupling constants of the connected protons (Table 1, Figures S3 and S4), indicated the presence of one trisubstituted double bond (*δ*_H_ 5.58, s, *δ*_C_ 130.4, CH; *δ*_C_ 136.5, qC) and four methyl groups (*δ*_H_ 0.97 (3H, d, Me-12); 0.90 (3H, d, Me-13); 1.40 (3H, s, Me-14); 1.42 (3H, t, Me-15)). The typical ^13^C NMR signal of sp^3^ quaternary carbon (*δ*_C_ 63.3, qC), bearing in mind the odd molecular weight of **1**, suggested the presence of an isocyano group (−NC group). The above functionalities account for three out of the five degrees of unsaturation, suggesting a bicyclic ring system in **1**. The above structural features were reminiscent of the co-occurring molecule **2**, as well as a previously reported axinisothiocyanate J (**20**) [46] which was isolated from the sponge *Axinyssa* sp.

**Figure 2:**
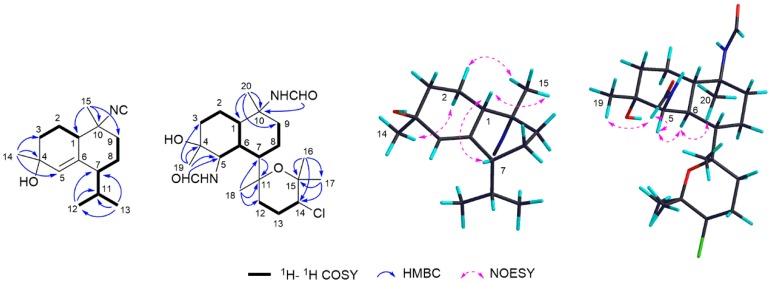
^1^H-^1^H COSY, key HMBC and NOESY correlations of compounds **1** and **16**.

Detailed comparison of the NMR data revealed that **1** should possess the same cadinane ring system as **20**. The only significant difference of these two compounds was the presence of an isocyano group at C-10 in **1** instead of the isothiocyano group (−NCS group) in **20**. According to this, the ^13^C NMR data of C-1, C-9, and C-10 in **1** were upfield shifted (*δ*_C_ 45.9, CH, Δ*δ* = −1.0 ppm; *δ*_C_ 39.4, CH_2_, Δ*δ* = −1.0 ppm; *δ*_C_ 63.3, qC, Δ*δ* = −2.7 ppm), respectively, compared with those in **20**. Further 2D NMR spectra, including COSY, HSQC, and HMBC (Figures S5–S7), allowed the unambiguous determination of the planar structure of compound **1** (Figure 2).

The relative configuration of **1** was deduced by NOESY spectra (Figure 2 and Figure S8). The NOE correlation between H-5 (*δ*_H_ 5.58, s) and H-11 (*δ*_H_ 2.14, m) indicated the *Z*-geometry of Δ^5,6^. The correlations of H-1 (*δ*_H_ 1.96, m) with Me-15 (*δ*_H_ 1.42, t) and H-7 (*δ*_H_ 1.64, m) indicated that these protons were on the same side of the molecule and were tentatively assigned to be *α*-oriented. Furthermore, the obvious NOE correlation between Me-15 and H-2b (1.94, m), and between Me-14 (1.40, s) and H-2a (1.69, m) suggested the *α*-orientation of Me-14. Therefore, the structure of compound **1** was determined as shown in Figure 1, which was further confirmed by its similar NMR data to those of axinisothiocyanate J (**20**) based on a biogenetic consideration [46]. In fact, compound **1** was identified as a C-10 epimer of a known isocyanosesquiterpene alcohol, which was first isolated from the nudibranch *Phyllidia pustulosa* [12].

### 2.2. Phyllidia coelestis

The abovementioned usual workup of the Et_2_O-soluble portion of the acetone extract of the animals of *P. coelestis* yielded six pure compounds: **6**, **8**–**11**, and **14** (Figure 1). The known compounds were identified as one eudesmane-type sesquiterpenoid: **6** [39,40], four bisabolane-type sesquiterpenoids: **8** [1], theonellin formamide (**9**) [33], theonellin isothiocyanate (**10**) [33], and 7-isocyano-7,8-dihydro-*α*-bisabolene (**11**) [42], and one mixture of pupukeanane-type sesquiterpenoids: **14** [6] by direct comparison of its NMR data and specific rotation with those reported in the literature.

### 2.3. Acanthella cavernosa

The frozen *A. cavernosa* animals were cut into pieces and exhaustively extracted by acetone. The Et_2_O-soluble portion of the acetone extract was repeatedly chromatographed to yield pure compounds **4**, **5**, **15**, **16**, **18**, and **19** (Figure 1). The known compounds were readily identified as one cadinane-type sesquiterpenoid: 10-formamido-4-cadinene (**4**) [24], one eudesmane-type sesquiterpenoid: axiriabiline A (**5**) [32], one spiroaxane-type sesquiterpenoid: axamide-3 (**15**) [27], along with two kalihinane-type diterpenoids: 10*β*-formamido-5*β*-isothiocyanatokalihinol-A (**18**) [14] and 10*β*-formamido-5-isocyanatokalihinol-A (**19**) [14] by comparing their NMR spectroscopic data and specific optical rotation with those reported in the literature.

Compound **16** was isolated as an optically active colorless oil, ${\left[\alpha \right]}_{D}^{20}$ +19 (*c* 0.1, CHCl_3_). Its molecular formula was determined as C_22_H_37_N_2_O_4_Cl by HRESIMS (*m*/*z* 429.2522 [M+H]^+^, calcd. 429.2515), indicating five degrees of unsaturation (Figure S9). The IR spectrum (Figure S10) of **16** showed absorptions at ν_max_ 1665 cm^−1^ and 3440 cm^−1^, indicating the presence of the amide carbonyl and hydroxy groups, respectively. The ^13^C NMR and DEPT spectra of **16** displayed 22 carbon signals, including five sp^3^ methyls, six sp^3^ methylenes, five sp^3^ methines, four sp^3^ quaternary carbons, and two sp^2^ methines. The spectroscopic data (Table 1, Figures S11 and S12) showed highly similarity to those of co-occurring related known compounds **18** and **19**, indicating that **16** is also a kalihinane-type diterpenoid. In fact, they differed from each other only by the substitution at C-5 position of the kalihinane ring. Bearing in mind the two additional protons present in its molecular formula in comparison to **19**, a −NHCHO group (*δ*_H_ 8.10 s, *δ*_C_ 167.6, CH) should be attached to the C-5 of compound **16**. Intriguingly, resonances for both formamides were observed as a plethora of signals between *δ*_H_ 8.0 and 8.3. These included eight signals arising from the four isomeric arrangements possible for the two formamides at C-5 and C-10 [47]. Detailed analysis of the 1D and 2D NMR spectra, including ^1^H-^1^H COSY, HSQC, and HMBC (Figures S13–S15), allowed the establishment of the planar structure of **16** (Figure 2), the same as a known compound named bisformamidokalihinol A, which was obtained from the hydrolysis of kalihinol A with acetic acid [48].

The relative configuration of **16** was also determined to be the same as co-occurring compounds **17**–**19** by careful interpretation of its NOESY spectrum with the clear NOE correlations of H-1/H-7, H-5/H-6/H_3_-20, and H_3_-19/NHCHO at C-5 (Figure 2 and Figure S16). Since the absolute configuration of **17** has been previously determined by total synthesis [29], from a biogenetic point of view, the absolute configuration of compound **16** was tentatively assigned as 1*S*,4*R*,5*R*,6*S*,7*S*,10*S*,11*R*,14*S*.

**Figure 3:**
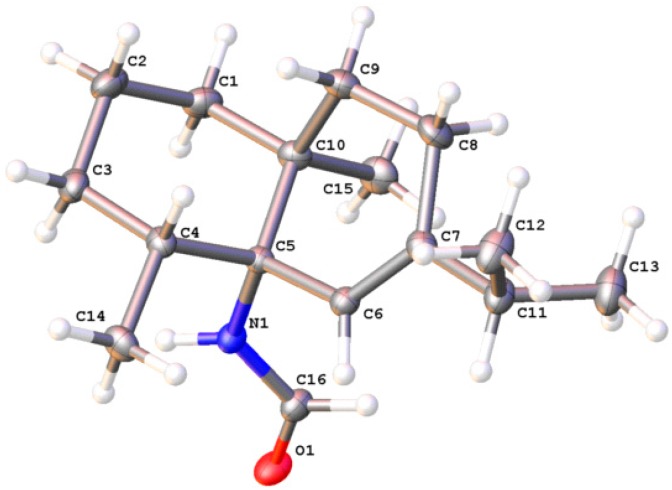
Perspective Oak Ridge Thermal Ellipsoid Plot (ORTEP) drawing of the X-ray structure of **5**.

It is worth noting that compound **5** was previously isolated from the Hainan sponge *Axinyssa variabilis*, and its absolute configuration was determined by a combination of ROESY experiment and time dependent density functional theory-electronic circular dichroism (TDDFT-ECD) calculation [32]. In this work, we obtained a single crystal of **5**, and X-ray diffraction (XRD) analysis on a suitable crystal of **5** by employing Ga K*α* radiation (*λ* = 1.34139 Å) with small Flack parameter 0.02 (16) allowed not only the unambiguous definition of the planar structure as illustrated in Figure 3, but also the revision of its absolute configuration from 4*S*,5*R*,10*S* to 4*R*,5*S*,10*S*.

Aware of the potent cytotoxicity exhibited by marine nitrogenous terpenoids, we performed in vitro biological evaluation of all the isolated metabolites on several tumor cell lines. The results (Table 2) showed that compounds **8**, **10**, and **11** exhibited strong cytotoxicity against human cancer cell line SNU-398 with IC_50_ values of 0.50, 2.15, and 0.50 μM, respectively. In addition, compound **8** also displayed broad cytotoxicity against the other three cancer cell lines, including A549, HT-29, and Capan-1, with IC_50_ values of 8.60, 3.35, and 1.98 μM, respectively. It is interesting to note that, although only three compounds showed cytotoxicity, they are all of the same bisabolane type. Therefore, a preliminary structure-activity relationship could be addressed, that is, the bisbolane skeleton might be good for activity, while regarding the inactive compounds **7** and **9**, the terminal olefin or the formamide group might be harmful for activity. More diverse bisabolanes should be discovered and tested for cytotoxicity to support our proposal.

## 3. Discussion

**Figure 4:**
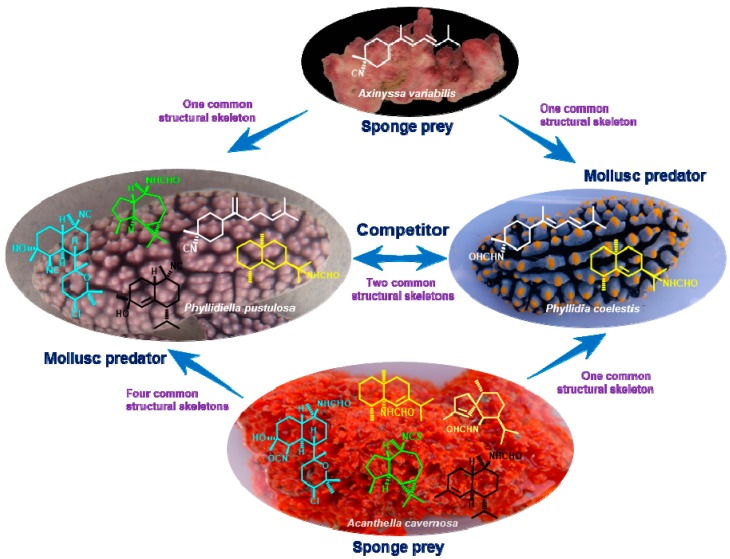
The common structural skeletons of the nudibranchs and their sponge-preys.

In recent years, several marine molluscs were found by our group to contain the same or similar secondary metabolites as those in marine corals or sponges, which was further proved to be due to the predator–prey relationship between these animals. For example, isoquinolinequinones were discovered from both the nudibranch *Jorunna funebris* and its sponge-prey *Xestospongia* sp. [49,50], while cladiellane-type diterpenoids were isolated from both the nudibranch *Tritoniopsis elegans* and its soft coral prey *Cladiella krempfi* [51]. In this study, similar results were observed by the chemical investigation of the three title animals. As shown in Figure 4, by comparison of the typical nitrogenous terpenoids in the two nudibranchs *P. pustulosa* and *P. coelestis* with those in the sponge *A. cavernosa*, four common structural skeletons were observed in both *P. pustulosa* and *A. cavernosa*, including cadinane, eudesmane, aromadendrane, and kalihinane, whereas one common eudesmane skeleton was found in all three animals. In addition, our previous chemical investigation of the marine sponge *A. variabilis* from the same water area in the South China Sea revealed the main secondary metabolites as bisabolene sesquiterpenoids [52], which was the common skeleton found in both *P. pustulosa* and *P. coelestis* (Figure 4). Therefore, on the basis of these research observations, we hold the belief that the two nudibranchs *P. pustulosa* and *P. coelestis* feed on the sponges *A. cavernosa* and *A. variabilis* and accumulate the useful dietary metabolites from the sponges, especially those toxic isocyanide derivatives, to be employed as their own chemical defensive agents for surviving in the harsh marine living environment. More intriguingly, it is obvious that one nudibranch can feed on various sponges to obtain diverse isocyanide metabolites, so as to use them as specially appointed chemical weapons on particular occasions.

In summary, the chemical investigation of the two nudibranchs *P. pustulosa* and *P. coelestis*, as well as the sponge *A. cavernosa*, led to the isolation and determination of 19 nitrogenous terpenoids with high chemical diversity. In fact, a total of seven different chemical skeletons were observed: four cadinane-type sesquiterpenoids (**1**–**4**), two eudesmane-type sesquiterpenoids (**5**–**6**), five bisabolene-type sesquiterpenoids (**7**–**11**), two aromadendrane-type sesquiterpenoids (**12** and **13**), one pupukeanane-type sesquiterpenoid (**14**), one spiroaxane-type sesquiterpenoid (**15**), and four kalihinane-type diterpenoids (**16**–**19**). Their structures including relative stereochemistry were elucidated by comprehensive NMR analyses. The absolute configuration of two new metabolites (**1** and **16**) were tentatively assigned based on the biogenetic consideration, whereas that of the known compound **5** was revised by the XRD analysis. In bioassay, the bisabolane-type sesquiterpenoids **8**, **10**, and **11** displayed considerable cytotoxicity against several cancer cell lines, which is worth further pharmacological study. Further chemical ecological research on the basis of the predator–prey relationship to prove our hypothesis would be interesting to be conducted in the future.

## 4. Materials and Methods

### 4.1. General Experimental Procedures

Optical rotations were measured in CHCl_3_ on a Perkin-Elmer 241MC polarimeter (PerkinElmer Inc., Waltham, MA, USA). IR spectra were recorded on a Nicolet 6700 spectrometer (Thermo Scientific, Waltham, MA, USA) with KBr pellets; peaks are reported in cm^−1^. 1D and 2D NMR spectra were measured on a Bruker DRX-400 or Bruker DRX-500 spectrometer (Bruker Biospin AG, Fällanden, Germany), using the residual CHCl_3_ signal (*δ*_H_ 7.26 ppm) as an internal standard for ^1^H NMR and CDCl_3_ (*δ*_C_ 77.00 ppm) for ^13^C NMR. Chemical shifts are expressed in *δ* (ppm) and coupling constants (*J*) in Hz. ^1^H and ^13^C NMR assignments were supported by ^1^H–^1^H COSY, HSQC, HMBC, and NOESY experiments. EIMS and HREIMS spectra were recorded on a Finnigan-MAT-95 mass spectrometer (FinniganMAT, San Jose, CA, USA). HRESIMS spectra were recorded on an Agilent G6250 Q-TOF (Agilent, Santa Clara, CA, USA). Reversed-phase (RP) HPLC purification was carried out on an Agilent 1260 series liquid chromatography equipped with a DAD G1315D detector at 210 and 254 nm and with a semi-preparative ODS-HG-5 column (5 μm, 250 × 9.4 mm). Commercial silica gel (Qingdao Haiyang Chemical Group Co., Ltd., Qingdao, China, 200–300 and 300–400 mesh) was used for column chromatography, and precoated silica gel plates (Yan Tai Zi Fu Chemical Group Co., Yantai, China, G60 F-254) were used for analytical Thin-layer chromatography (TLC). Spots were detected on TLC under UV light or by heating after spraying with anisaldehyde H_2_SO_4_ reagent. All the chemicals were obtained from commercial sources. All solvents used for column chromatography (CC) were of analytical grade, and solvents used for HPLC were of HPLC grade.

### 4.2. Biological Material, Extraction, and Isolation

#### 4.2.1. Biological Material

The molluscs and sponges were collected using scuba at Xidao Island, Hainan Province, China, in March 2014, at a depth of −15 to −20 m, and identified by Professor Xiu-Bao Li from Hainan University. The voucher sample is deposited at the Shanghai Institute of Materia Medica, CAS.

#### 4.2.2. Extraction and Isolation of **1**–**19**

The lyophilized bodies of *P. pustulosa* (24 specimens, 11.1 g, dry weight) were carefully dissected into internal organs and mantle that were separately extracted by acetone using ultrasound. Filtration of the two homogenates gave an aqueous-Me_2_CO filtrate that was concentrated in vacuo to give a gummy residue. The residue was suspended in H_2_O and extracted sequentially with diethyl ether and *n*-BuOH. The mantle ether extract (431.3 mg) was subjected to a silica gel column eluting with light petroleum ether/diethyl ether gradient to yield 11 fractions (A-K), including pure compounds **3** (5.3 mg), **12** (2.6 mg), and **13** (1.0 mg). A less polar fraction E was chromatographed over Sephadex LH-20 eluting with PE/CHCl_3_/MeOH (2:1:1), followed by silica gel CC (PE/Et_2_O, 100:1 to 50:1) to afford **7** (2.0 mg), **8** (2.2 mg), and **14** (2.6 mg). A middle polar fraction I was separated by a column of Sephadex LH-20 eluting with CHCl_3_/MeOH (1:1), followed by ODS CC (MeOH/H_2_O, 60:40) to afford **1** (1.5 mg) and **2** (1.0 mg). Fraction J was chromatographed over Sephadex LH-20 eluting with CHCl_3_/MeOH (1:1), followed by silica gel CC (PE/Et_2_O, 6:4), and was further purified by ODS CC (MeOH/H_2_O, 50:50) to yield **5** (2.0 mg) and **17** (3.1 mg). The digestive gland ether extract (60.0 mg) was purified by a silica gel column eluting with light petroleum ether/diethyl ether gradient, followed by a similar procedure as above, to give compounds **3** (1.3 mg), **5** (0.5 mg), **7** (1.3 mg), **8** (1.8 mg), **14** (0.9 mg), and **17** (1.9 mg).

The lyophilized bodies of *P. coelestis* (seven specimens, 25.5 g, dry weight) were extracted by acetone using ultrasound. The extracts of both internal organs and mantle were combined due to the similar TLC results, to give 700 mg extract. An approach similar to the abovementioned fractional method was applied to give a total of seven fractions (A–G). Compounds **8** (5.2 mg) and **9** (3.4 mg) were obtained directly from fractions B and G after purification by HPLC, respectively. Fraction B was chromatographed over Sephadex LH-20 eluting with PE/CHCl_3_/MeOH (2:1:1), followed by HPLC purification to give compounds **10** (1.5 mg) and **11** (1.2 mg). Fraction F was treated by the same procedure as above to give compound **6** (1.7 mg).

The frozen *A. cavernosa* sponges (55 g, dry weight) were cut into pieces and extracted exhaustively with acetone at room temperature (6 × 2.0 L). The organic extract was evaporated to give a brown residue, which was then partitioned between H_2_O and Et_2_O. The upper layer was concentrated under reduced pressure to give a red residue (1.0 g). The resultant residue was separated into six fractions (A–F) by gradient silica gel column chromatography. The resulting fractions were then fractionated into sub-fractions by Sephadex LH-20. The sub-fraction F6 was purified by semi-preparative HPLC (70% MeOH to 100% MeOH in 20 min), yielding compounds **16** (4.0 mg), **18** (2.0 mg), and **19** (1.9 mg). The sub-fraction E4 of fraction E gave compounds **4** (3.1 mg), **6** (4.1 mg), and **15** (2.7 mg).

*Xidaoisocyanate A* (**1**), colorless oil, ${\left[\alpha \right]}_{D}^{20}$ −3.6 (*c* 0.1, MeOH); for ^1^H and ^13^C NMR spectroscopic data, see Table 1; HREIMS: *m*/*z* calcd for C_16_H_25_NO [M]^+^: 247.1936; found: 247.1927.

*Bisformamidokalihinol A* (**16**), colorless oil, ${\left[\alpha \right]}_{D}^{20}$ +19 (*c* 0.1, CHCl_3_); for ^1^H and ^13^C NMR spectroscopic data, see Table 1; HRESIMS: *m*/*z* calcd for C_22_H_3__8_N_2_O_4_Cl [M+H]^+^: 429.2515; found: 429.2522.

*Axiriabiline A* (**5**), colorless crystal, m.p. 105−107 °C, ${\left[\alpha \right]}_{D}^{20}$ −123 (*c* 0.1, CHCl_3_); X-ray crystal data for compound **5**: C_16_H_27_NO *M* = 249.38, orthorhombic, *a* = 11.5594(2) Å, *b* = 12.0694(2) Å, *c* = 21.2049(4) Å, *α* = 90.00°, *β* = 90.00°, *γ* = 90.00°, *V* = 2958.40(9) Å^3^, *T* = 170.01 K, space group *P*2(1)2(1)2(1), *Z* = 8, 28095 reflections measured, 5616 independent reflections (*R_int_* = 0.0569). The final *R_1_* values were 0.0416 (*I* > 2*σ*(*I*)). The final *wR*(*F*^2^) values were 0.1051 (*I* > 2*σ*(*I*)). The final *R_1_* values were 0.0446 (all data). The final *wR*(*F*^2^) values were 0.1081 (all data). The structure was solved by direct methods (SHELXS97) and refined using full-matrix least-squares difference Fourier techniques. All non-hydrogen atoms were refined anisotropically, and all hydrogen atoms were placed in idealized positions and refined as riding atoms with their related isotropic parameters. Crystallographic data (excluding structure factors) for the structure in this paper have been deposited with the Cambridge Crystallographic Data Center as supplementary publication no. CCDC 1880256. Copies of the data can be obtained, free of charge, on application to CCDC, 12 Union Road, Cambridge CB2 1EZ, UK (fax: +44-(0)1223-336033 or e-mail: deposit@ccdc.cam.ac.uk).

### 4.3. Bioassay Procedures

#### Cytotoxic Activity

Compounds **1**–**19** were evaluated for their cytotoxic activity against four human cancer cell lines (A549, HT-29, SNU-398, and Capan-1) using the sulforhodamine B (SRB, Sigma, St. Louis, MO, USA) method. Four cell lines were purchased from the American Type Culture Collection (ATCC, Manassas, VA, USA). The cytotoxic activity in vitro was indicated in terms of IC_50_ (μM), that is, the concentration of a compound that inhibited the proliferation rate of tumor cells by 50% as compared to the untreated control cells. Vincristine was used as a reference drug.

## Figures and Tables

**Table 1:** ^1^H and ^13^C NMR data of **1** and **16**, and their model compounds **20** and **17**, respectively, recorded in CDCl_3_ *^a^*.

No.	1		20	No.	16		17
	*δ*_H_ Mult (*J* in Hz)	*δ*_C_	*δ*_C_		*δ*_H_ Mult (*J* in Hz)	*δ*_C_	*δ*_C_
1	1.96 m	45.9 CH	46.9 CH	1	1.37 m	42.6 CH	42.3 CH
2a	1.69 m	21.6 CH_2_	21.6 CH_2_	2a	1.47 m	21.7 CH_2_	21.6 CH_2_
2b	1.94 m			2b	1.61 m		
3a	1.53 m	36.3 CH_2_	36.2 CH_2_	3	1.51 m, 2H	33.7 CH_2_	32.6 CH_2_
3b	1.96 m						
4	-	69.5 qC	69.3 qC	4	-	71.6 qC	70.5 qC
5	5.58 s	130.4 CH	130.2 CH	5	4.18 (d, 10.4)	59.8 CH	63.7 CH
6	-	136.5 qC	137.2 qC	6	2.35 m	36.6 CH	36.0 CH
7	1.64 m	47.3 CH	47.4 CH	7	1.57 m	45.8 CH	48.4 CH
8a	1.49 m	21.8 CH_2_	22.5 CH_2_	8a	1.62 m	23.1 CH_2_	21.9 CH_2_
8b	1.69 m			8b	1.02 m		
9a	1.51 m	39.4 CH_2_	40.4 CH_2_	9a	1.72 m	40.7 CH_2_	39.7 CH_2_
9b	2.01 (d, 10.0)			9b	1.55 m		
10	-	63.3 qC	66.0 qC	10	-	55.0 qC	59.0 qC
11	2.14 m	26.8 CH	26.8 CH	11	-	79.0 qC	76.8 qC
12	0.97 (d, 6.8)	22.1 CH_3_	22.1 CH	12a	1.48 m	38.1 CH_2_	38.0 CH_2_
				12b	1.57 m		
13	0.90 (d, 6.8)	17.5 CH_3_	17.5 CH	13a	1.99 m	27.7 CH_2_	27.4 CH_2_
				13b	2.06 m		
14	1.40 s	27.1 CH_3_	26.7 CH_3_	14	3.68 (dd, 12.4, 4.4)	64.4 CH	64.1 CH
15	1.42 (t, 1.8)	28.9 CH_3_	28.2 CH_3_	15	-	76.7 qC	76.0 qC
NC (**1**) and NCS (**20**)	-	n.d. *^b^*	n.d. *^b^*	16	1.37 s	23.5 CH_3_	22.8 CH_3_
				17	1.31 s	31.4 CH_3_	30.5 CH_3_
				18	1.27 s	19.7 CH_3_	19.2 CH_3_
				19	1.19 s	18.8 CH_3_	29.0 CH_3_
				20	1.18 s	29.0 CH_3_	20.7 CH_3_
				CHO-1 or NC	8.25 (d, 12.0)	163.7 CH	157.0 qC
				CHO-2 or NC	8.10 (d, 11.4)	167.6 CH	153.0 qC

*^a^* Assignments were deduced by the analysis of 1D and 2D NMR spectra. *^b^* n.d. means not detected.

**Table 2:** Cytotoxicity of compounds **1**–**19** against four human cancer cell lines.

Compounds *^a^*	A549	HT-29	SNU-398	Capan-1
	IC_50_ (μM)			
**8**	8.60 ± 6.36	3.35 ± 3.12	0.50 ± 0.46	1.98 ± 1.76
**10**	>50	>50	2.15 ± 0.93	>50
**11**	>50	>50	0.50 ± 0.35	>50
**VCR**	10.13 nM	0.23 nM	0.04 nM	0.30 nM

^*a*^ Compounds **1**–**7**, **9**, **12**–**19** were considered to be inactive with IC_50_ values of more than 50 μM; VCR: vincristine.

## Supplementary Materials

The following are available online at http://www.mdpi.com/1660-3397/17/1/56/s1, Figure S1. LREIMS spectrum of compound **1**. Figure S2. HREIMS spectrum of compound **1**. Figure S3. ^1^H NMR spectrum of compound **1** in CDCl_3_. Figure S4. ^13^C NMR spectrum of compound **1** in CDCl_3_**. Figure S5**. HSQC spectrum of compound **1** in CDCl_3_. Figure S6. ^1^H-^1^H COSY spectrum of compound **1** in CDCl_3_. Figure S7. HMBC spectrum of compound **1** in CDCl_3_. Figure S8. NOESY spectrum of compound **1** in CDCl_3_. Figure S9. HRESIMS spectrum of compound **16**. Figure S10. IR spectrum of compound **16**. Figure S11. ^1^H NMR spectrum of compound **16** in CDCl_3_. Figure S12. ^13^C NMR spectrum of compound **16** in CDCl_3_. Figure S13. HSQC spectrum of compound **16** in CDCl_3_. Figure S14. ^1^H-^1^H COSY spectrum of compound **16** in CDCl_3_. Figure S15. HMBC spectrum of compound **16** in CDCl_3_. Figure S16. NOESY spectrum of compound **16** in CDCl_3_.
