# Supplementary material for: Cytotoxic Nitrogenous Terpenoids from Two South China Sea Nudibranchs Phyllidiella pustulosa, Phyllidia coelestis, and Their Sponge-Prey Acanthella cavernosa
Source: Mar Drugs. 2019 Jan 16;17(1):56. doi: 10.3390/md17010056 (PMC6356796; doi:10.3390/md17010056)
Supplement: Supplementary file 1 [file marinedrugs-17-00056-s001.pdf]

Supporting Information

Cytotoxic Nitrogenous Terpenoids from Two South China Sea Nudibranchs *Phyllidiella pustulosa, Phyllidia coelestis*, and Their Sponge-Prey *Acanthella cavernosa*

Qihao Wu [1,2,†], Wen-Ting Chen [1,†], Song-Wei Li [1], Jian-Yu Ye [1], Xia-Juan Huan [1], Margherita Gavagnin [3], Li-Gong Yao [1], Hong Wang [2], Ze-Hong Miao [1], Xu-Wen Li [1,*] and Yue-Wei Guo [1,2,*]

1 State Key Laboratory of Drug Research, Shanghai Institute of Materia Medica, Chinese Academy of Sciences, Zuchongzhi Road 555 Zhangjiang Hi-Tech Park, Shanghai 201203, China; qihaowu@zjut.edu.cn (Q.W.); windychen_simm@163.com (W.-T.C.); simmswli@163.com (S.-W.L.); 3150103114@zju.edu.cn (J.-Y.Y.); huanxj@simm.ac.cn (X.-J.H.); yaoligong@simm.ac.cn (L.-G.Y.); zhmiao@simm.ac.cn (Z.-H.M.)

2 College of Pharmaceutical Sciences, Zhejiang University of Technology, Hangzhou 310014, China; hongw@zjut.edu.cn

3 Consiglio Nazionale delle Ricerche (CNR), Istituto di Chimica Biomolecolare (ICB), Via Campi Flegrei, 34, 80078 Pozzuoli (Na), Italy; mgavagnin@icb.cnr.it

* Correspondence: xwli@simm.ac.cn (X.-W.L.); ywguo@simm.ac.cn (Y.-W.G.); Tel.: +86-21-50805813

Table of Contents

1. 1D, 2D NMR, MS and IR spectra of compounds **1** and **16**.. 3
    1.1 Spectra for xidaoisocyanate A (**1**) 3
    1.2 Spectra for bisformamidokalihinol A (**16**).. 7

1. 1D, 2D NMR, MS and IR spectra of compounds 1 and 16

1.1 Spectra for xidaoisocyanate A (1)

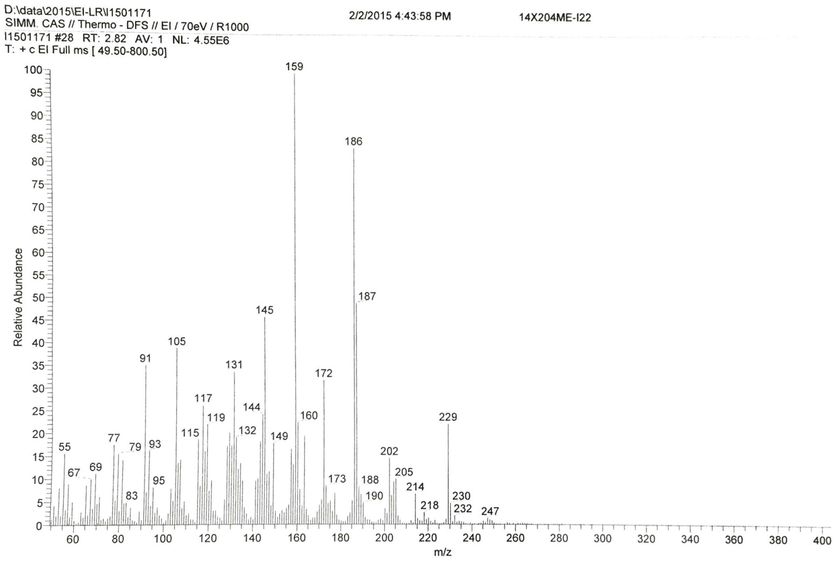

Figure S1. LREIMS spectrum of compound **1**

Mass		27350 Intensity	%RA	%RIC	(mmu) Delta	R+D	Composition
148.0854	*	40103	3.86	0.15			
149.0242	*	121093	11.66	0.46	2.3	11.0	C11.H3.N
149.0964	*	111620	10.74	0.42	0.2	4.5	C10.H13.O
149.1325	*	78924	7.60	0.30	0.5	3.5	C11.H17
150.0993	*	32552	3.13	0.12			
155.0853	*	30843	2.97	0.12	0.8	7.5	C12.H11
157.1000	*	218253	21.01	0.82	1.7	6.5	C12.H13
158.1015	*	138331	13.31	0.52			
159.0795	*	35330	3.40	0.13	1.5	6.5	C11.H11.O
159.1162	*	960203	92.42	3.62	1.1	5.5	C12.H15
160.1212	*	219536	21.13	0.83			
161.0945	*	64393	6.20	0.24	2.1	5.5	C11.H13.O
161.1293	*	65889	6.34	0.25			
162.0969	*	37040	3.57	0.14			
163.1112	*	123088	11.85	0.46	1.1	4.5	C11.H15.O
163.1475	*	260423	25.07	0.98	1.1	3.5	C12.H19
164.1132	*	29062	2.80	0.11			
164.1509	*	31983	3.08	0.12			
170.0978	*	31983	3.08	0.12	-0.8	7.5	C12.H12.N
171.1098	*	46229	4.45	0.17			
172.1139	*	250806	24.14	0.95	-1.2	6.5	C12.H14.N
173.0969	*	44875	4.32	0.17	-0.3	6.5	C12.H13.O
173.1168	*	29632	2.85	0.11			
173.1330	*	43380	4.18	0.16	0.0	5.5	C13.H17
174.1328	*	42881	4.13	0.16			
175.1126	*	136693	13.16	0.52	-0.3	5.5	C12.H15.O
175.1481	*	29561	2.85	0.11	0.6	4.5	C13.H19
176.1154	*	56130	5.40	0.21			
177.1278	*	93527	9.00	0.35	0.1	4.5	C12.H17.O
177.1605	*	39747	3.83	0.15			
178.1312	*	28065	2.70	0.11			
183.1166	*	30772	2.96	0.12	0.7	7.5	C14.H15
185.1203	*	33763	3.25	0.13	0.1	7.0	C13.H15.N
186.1276	*	1038914	100.00	3.92	0.7	6.5	C13.H16.N
187.1391	*	570993	54.96	2.15			
188.1393	*	45160	4.35	0.17			
188.1533	*	35330	3.40	0.13			
189.1574	*	77143	7.43	0.29			
190.1602	*	61045	5.88	0.23	-0.6	4.5	C13.H20.N
193.1215	*	29418	2.83	0.11			
200.1542	*	41955	4.04	0.16	2.3	6.0	C15.H20
201.1610	*	36114	3.48	0.14			
202.1236	*	33336	3.21	0.13	-0.4	6.5	C13.H16.N.O
202.1713	*	114041	10.98	0.43	0.9	5.0	C15.H22
203.1402	*	41812	4.02	0.16			
203.1790	*	68453	6.59	0.26	0.9	4.5	C15.H23
204.1400	*	124370	11.97	0.47	-1.1	5.5	C13.H18.N.O
204.1780	*	57911	5.57	0.22	-2.8	4.5	C14.H22.N
205.1598	*	164473	15.83	0.62	-0.5	4.5	C14.H21.O
214.1602	*	62897	6.05	0.24	-0.6	6.5	C15.H20.N
216.1513	*	27566	2.65	0.10	0.1	6.0	C15.H20.O
218.1667	*	69308	6.67	0.26	0.4	5.0	C15.H22.O
219.1759	*	50503	4.86	0.19	-1.0	4.5	C15.H23.O
229.1825	*	178791	17.21	0.67	0.5	6.0	C16.H23.N
230.1882	*	30344	2.92	0.11	2.7	5.5	C16.H24.N
232.1668	*	31270	3.01	0.12			
247.1927	*	27352	2.63	0.10	0.9	5.0	C16.H25.N.O

Figure S2. HREIMS spectrum of compound **1**

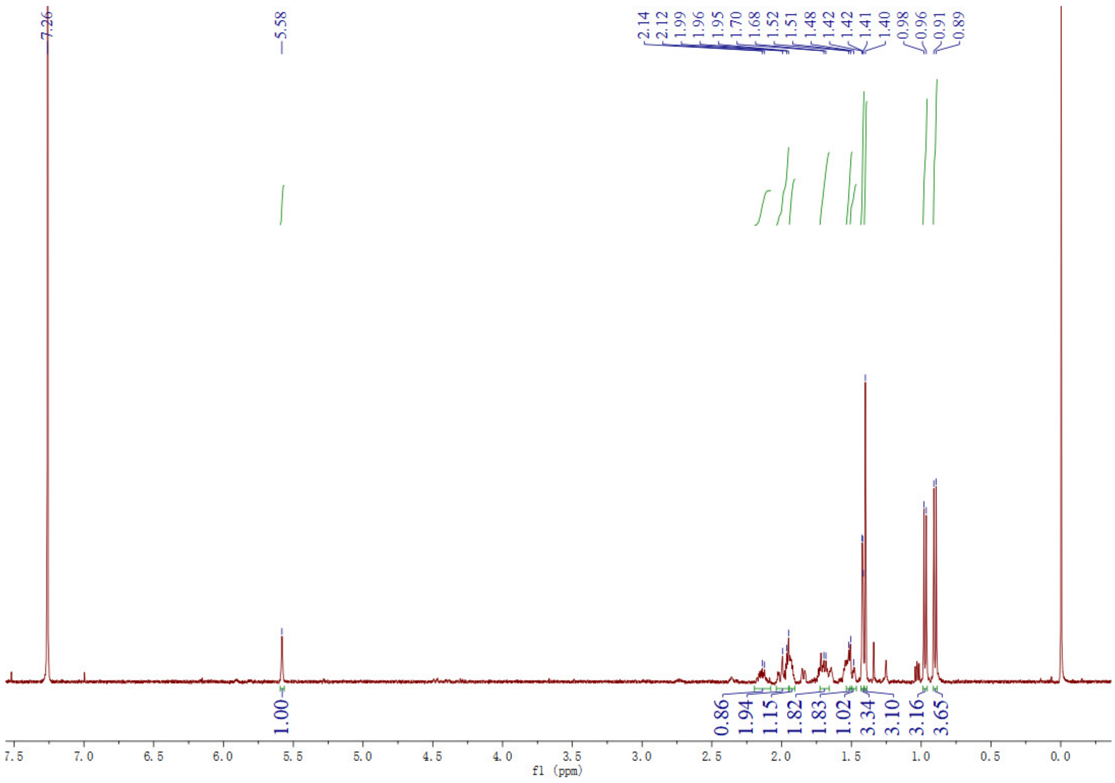

Figure S3. ^{1}H NMR spectrum of compound **1** in $CDCl_3$

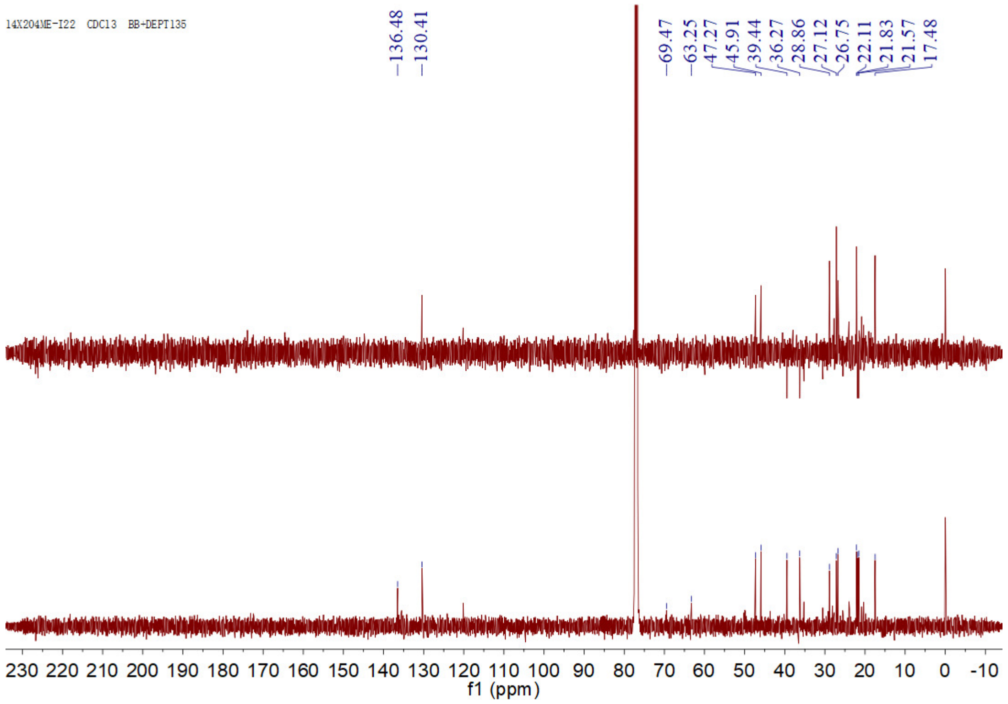

Figure S4. ^{13}C NMR spectrum of compound **1** in $CDCl_3$

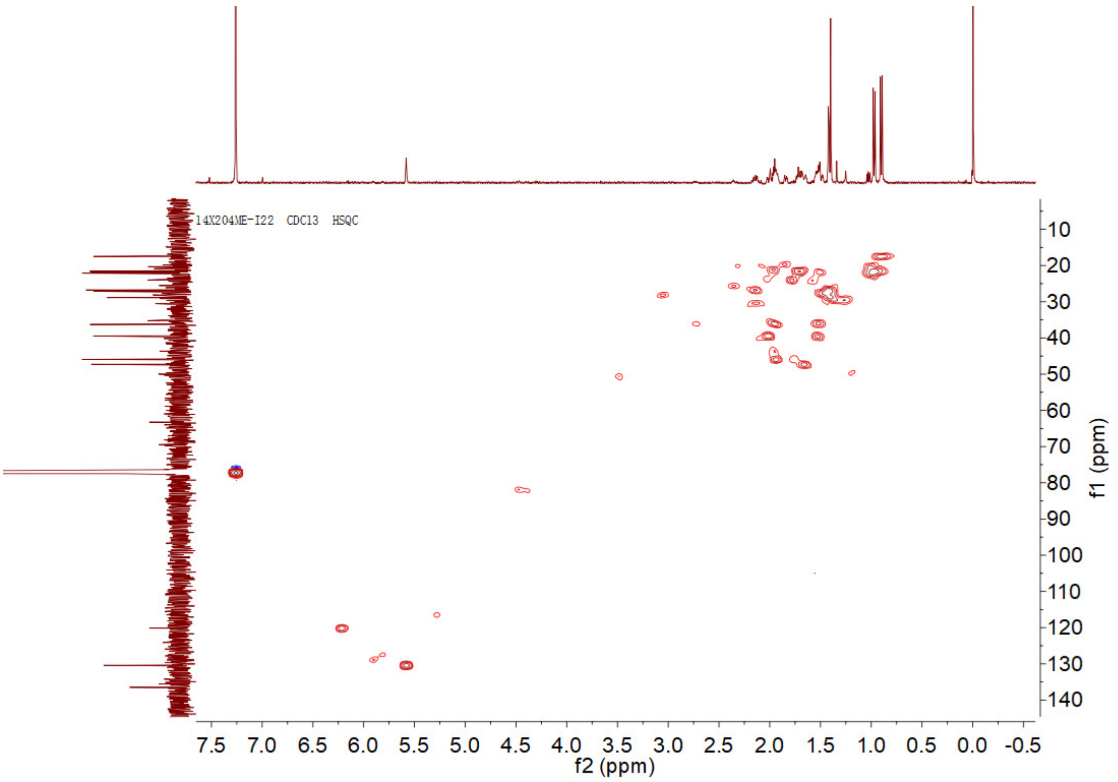

Figure S5. HSQC spectrum of compound **1** in $CDCl_3$

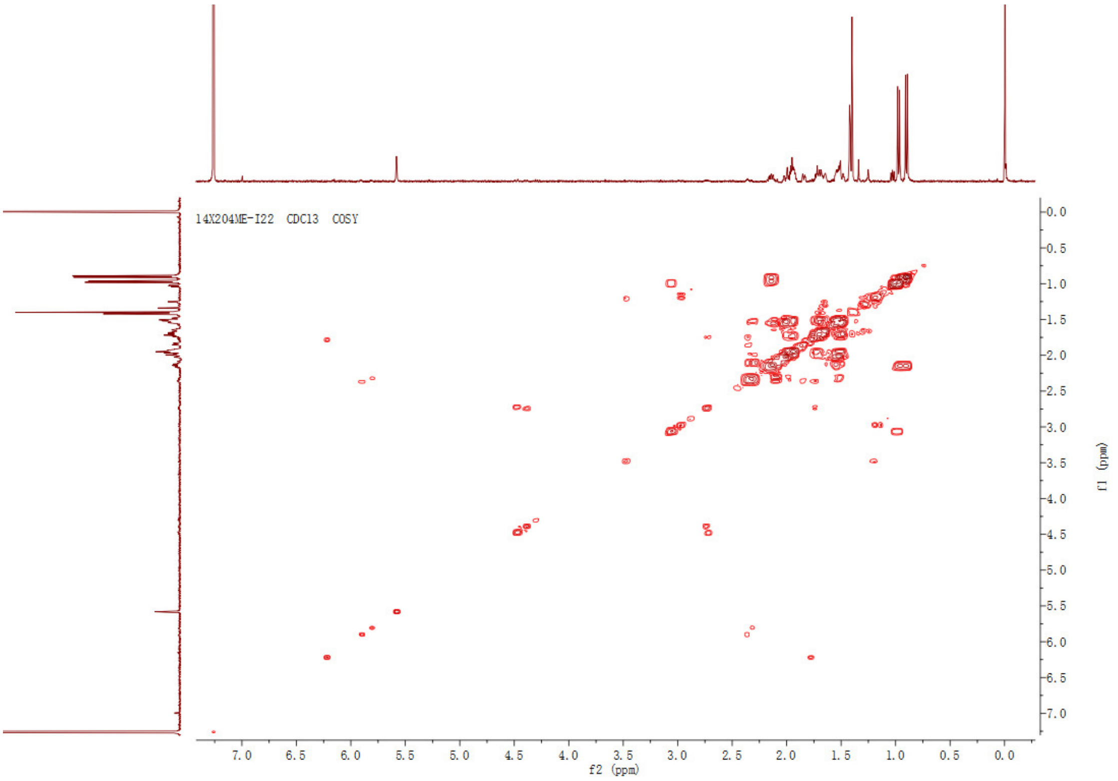

Figure S6. ^{1}H-^{1}H COSY spectrum of compound **1** in $CDCl_3$

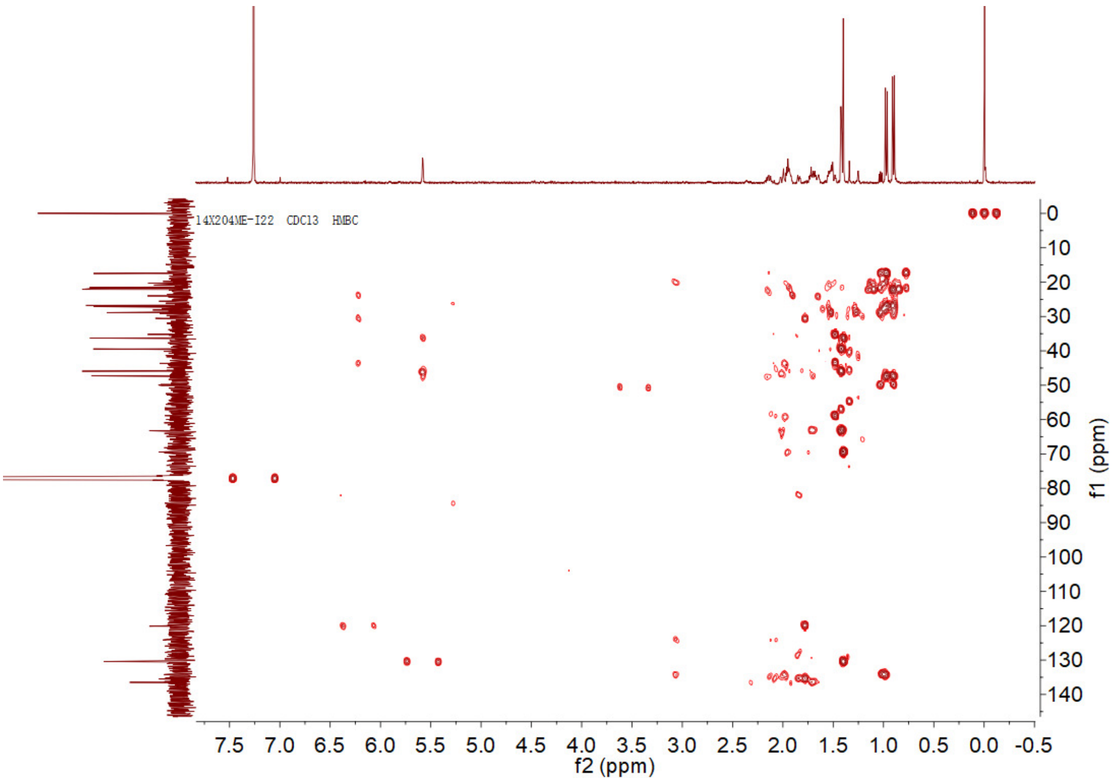

Figure S7. HMBC spectrum of compound **1** in $CDCl_3$

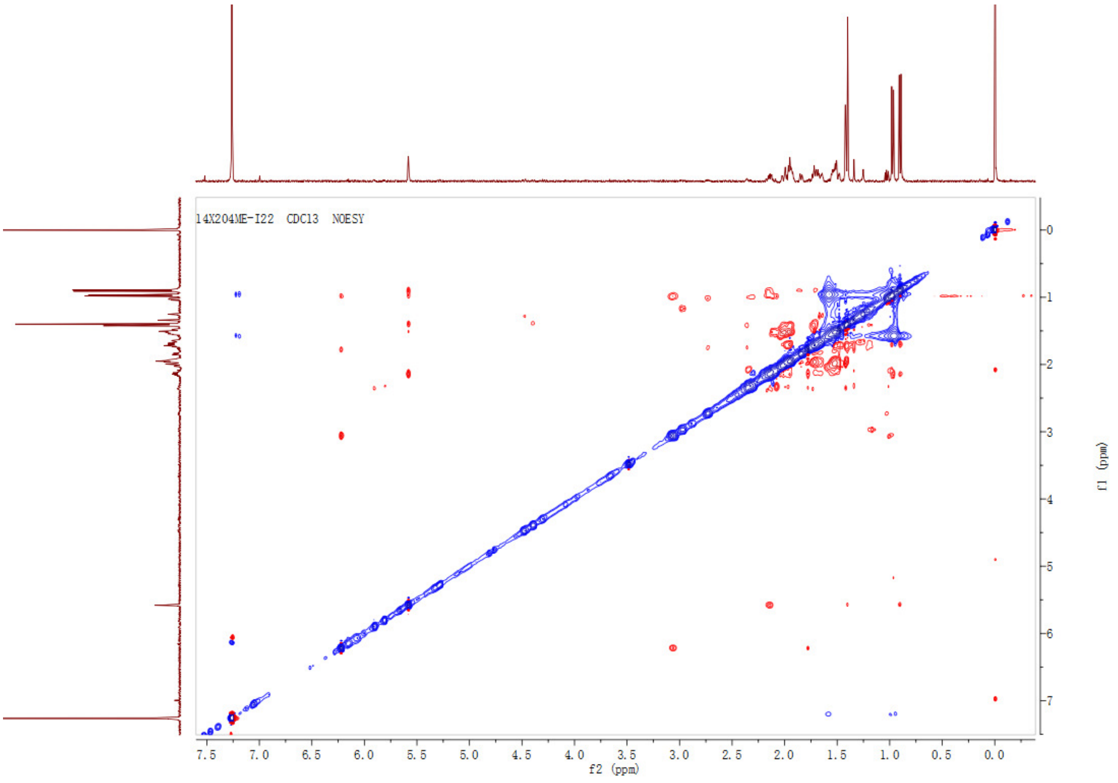

Figure 8. NOESY spectrum of compound **1** in $CDCl_3$

1.2 Spectra for bisformamidokalihinol A (16)

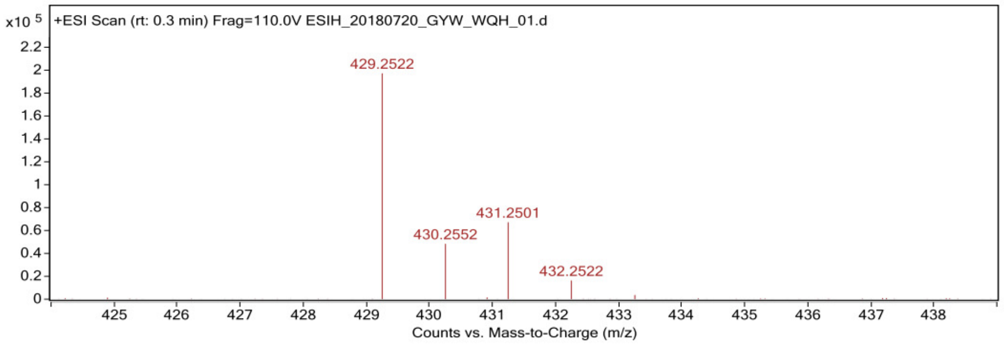

Formula Calculator Results

m/z	Calc m/z	Diff (mDa)	Diff (ppm)	Ion Formula	Ion
429.2522	429.2515	-0.73	-1.7	C22 H38 Cl N2 O4	(M+H)+

Figure S9. HRESIMS spectrum of compound **16**

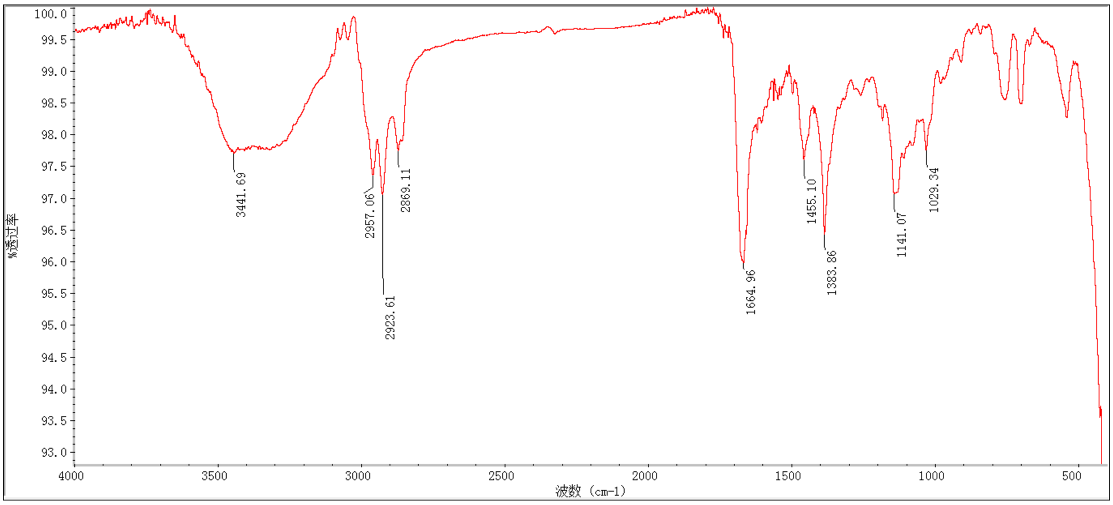

Figure S10. IR spectrum of compound **16**

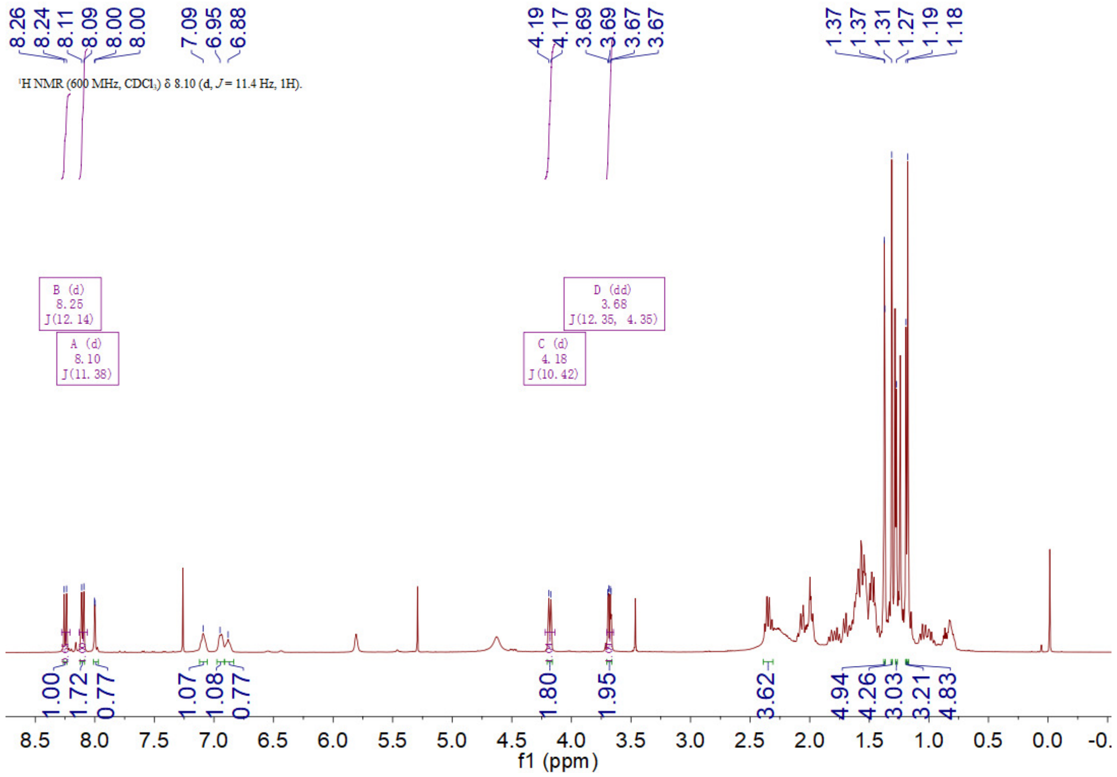

Figure S11. ^{1}H NMR spectrum of compound **16** in $CDCl_3$

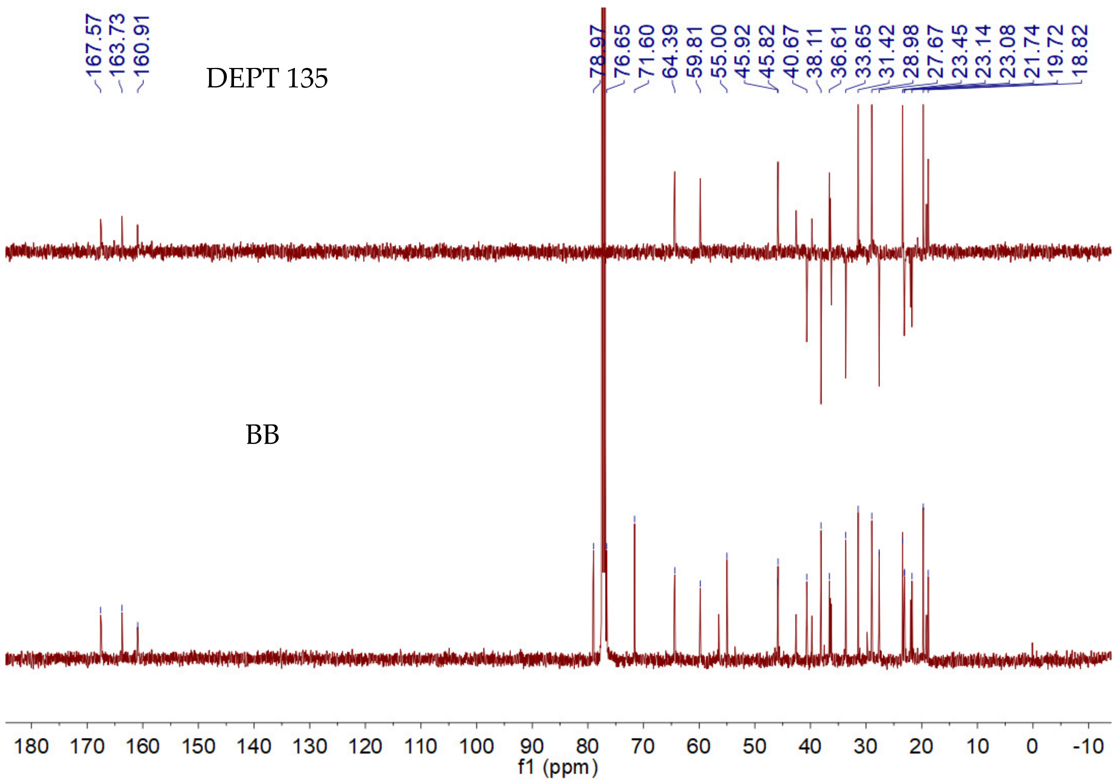

Figure S12. ^{13}C NMR spectrum of compound **16** in $CDCl_3$

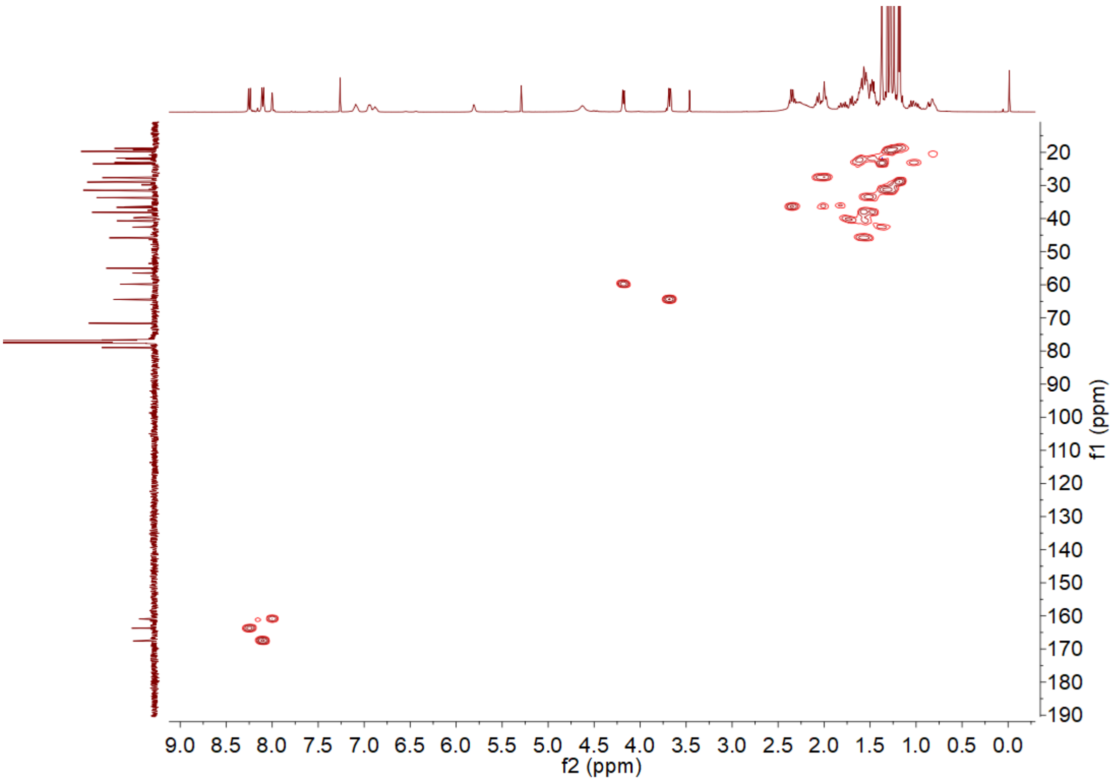

Figure S13. HSQC spectrum of compound **16** in $CDCl_3$

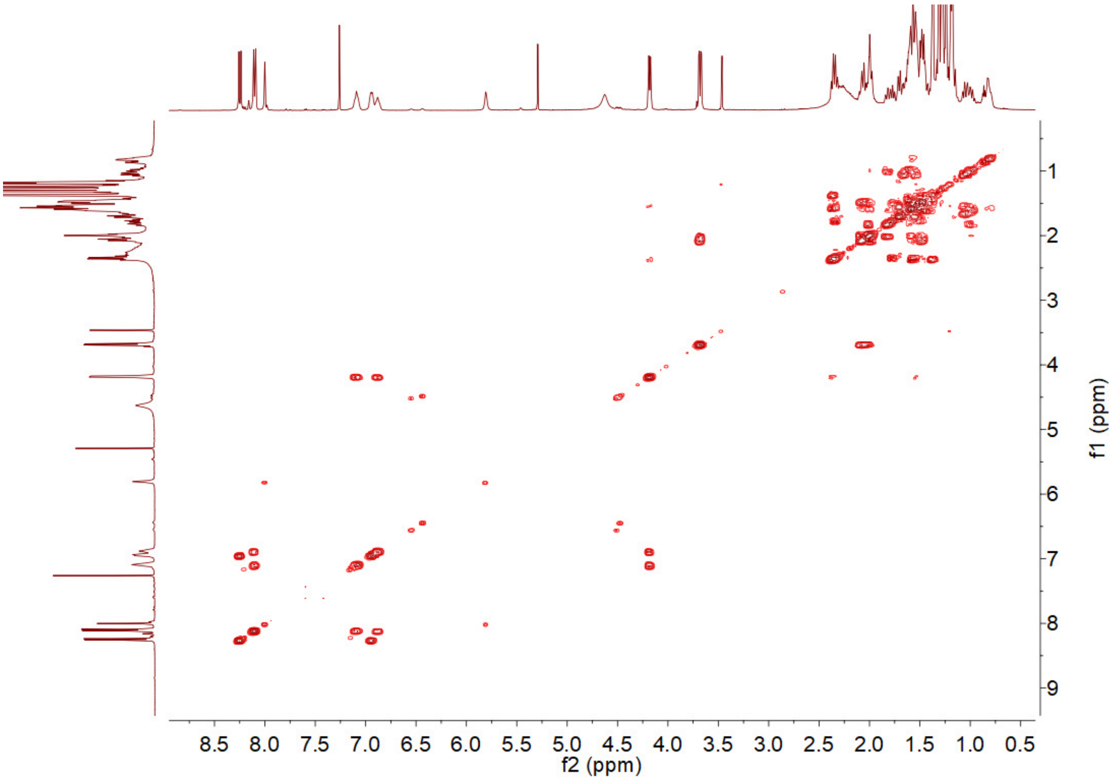

Figure S14. ^1H-^1H COSY spectrum of compound **16** in $CDCl_3$

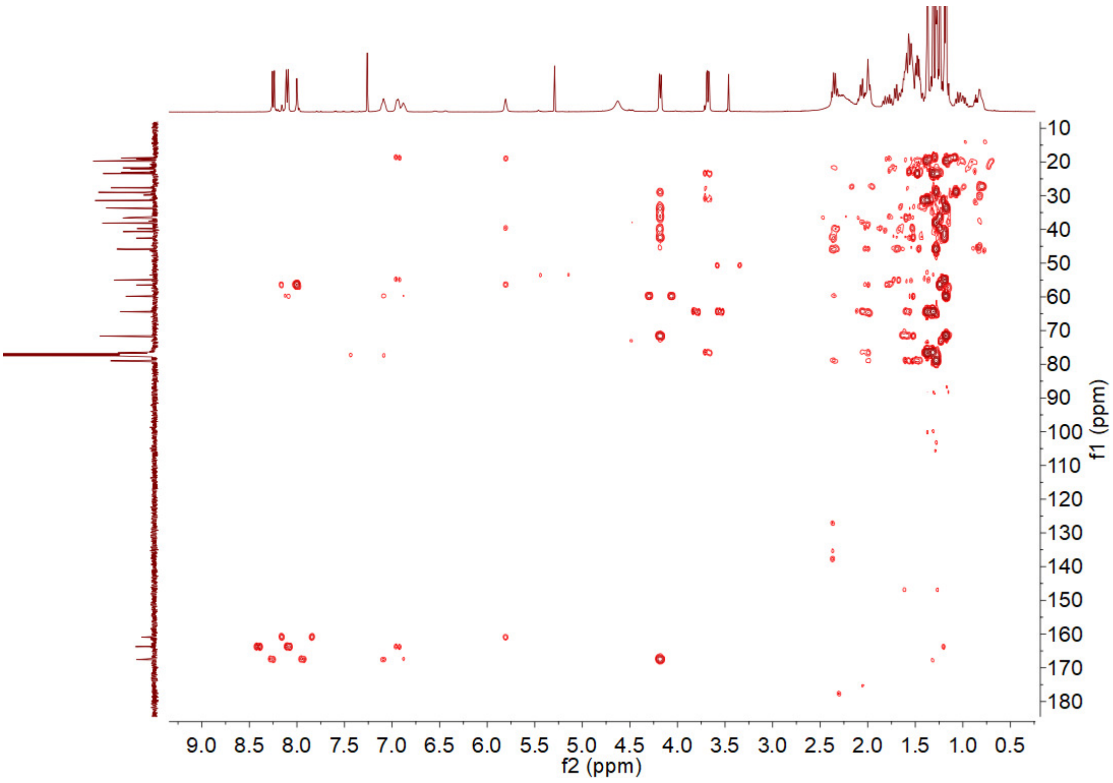

Figure S15. HMBC spectrum of compound **16** in $CDCl_3$

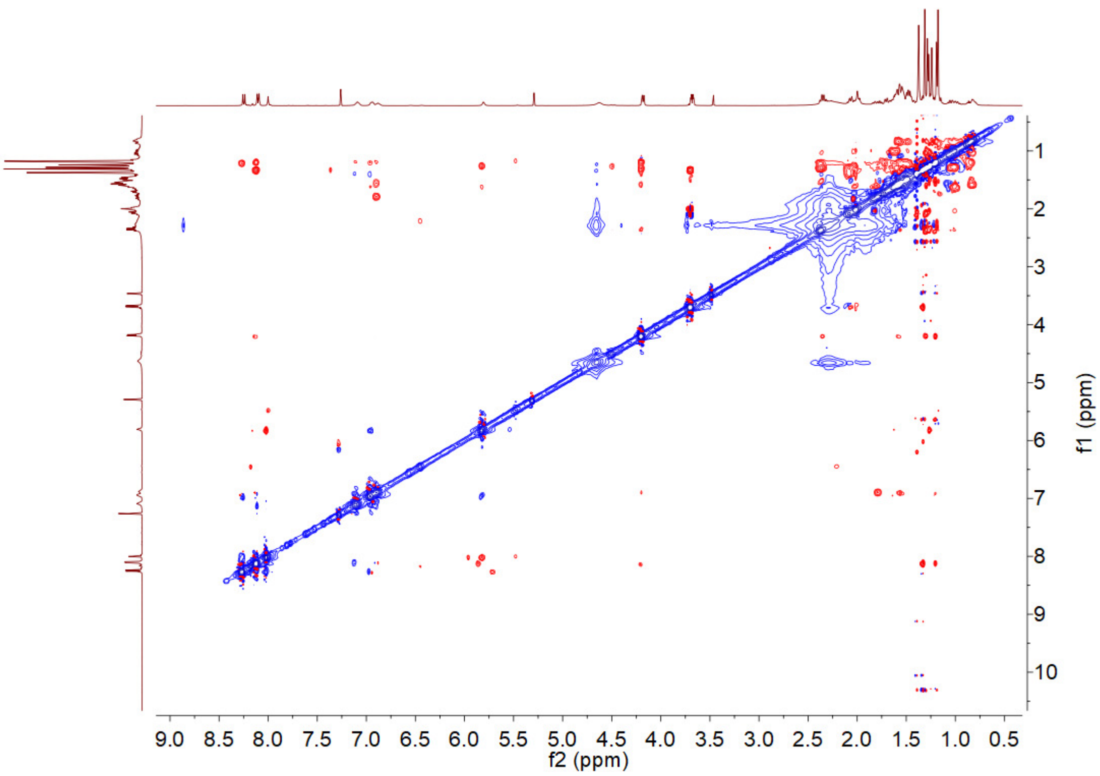

Figure S16. NOESY spectrum of compound **16** in $CDCl_3$
